# Metabolism Dysregulation in Retinal Diseases and Related Therapies

**DOI:** 10.3390/antiox11050942

**Published:** 2022-05-11

**Authors:** Yingying Chen, Nathan J. Coorey, Meixia Zhang, Shaoxue Zeng, Michele C. Madigan, Xinyuan Zhang, Mark C. Gillies, Ling Zhu, Ting Zhang

**Affiliations:** 1 Department of Ophthalmology, West China Hospital, Sichuan University, Chengdu 610017, China; 2019324020107@stu.scu.edu.cn; 2 Save Sight Institute, Faculty of Medicine and Health, The University of Sydney, Sydney, NSW 2000, Australia; shaoxue.zeng@sydney.edu.au (S.Z.); m.madigan@unsw.edu.au (M.C.M.); mark.gillies@sydney.edu.au (M.C.G.); ling.zhu@sydney.edu.au (L.Z.); 3 Canberra Health Services, Canberra, ACT 2605, Australia; nathan.coorey@gmail.com; 4 Macular Disease Research Laboratory, Department of Ophthalmology, West China Hospital, Sichuan University, Chengdu 610017, China; 5 School of Optometry and Vision Science, University of New South Wales (UNSW), Sydney, NSW 2052, Australia; 6 Department of Ocular Fundus Diseases, Beijing Tongren Eye Centre, Tongren Hospital, Capital Medical University, Beijing 100073, China; mmzxy2010@163.com; 7 Beijing Retinal and Choroidal Vascular Study Group, Beijing 100073, China

**Keywords:** retina, metabolism, retinal diseases, gene therapy, lipid metabolism

## Abstract

The human retina, which is part of the central nervous system, has exceptionally high energy demands that requires an efficient metabolism of glucose, lipids, and amino acids. Dysregulation of retinal metabolism disrupts local energy supply and redox balance, contributing to the pathogenesis of diverse retinal diseases, including age-related macular degeneration, diabetic retinopathy, inherited retinal degenerations, and Macular Telangiectasia. A better understanding of the contribution of dysregulated metabolism to retinal diseases may provide better therapeutic targets than we currently have.

## 1. Introduction

### 1.1. General Retinal Metabolism

The human retina is part of the central nervous system. Vascularised by two vascular beds (the retinal and choroidal vasculatures), the retina shares the characteristically high metabolism of the brain [1]. Exceptional energy expenditure is required to maintain resting photoreceptor membrane potential [2], replace photoreceptor outer segments [3], and combat retinal oxidative stress [4]. ATP, the currency of energy, is generated most efficiently from oxidative metabolism in the mitochondria [5]. Alternatively, phosphocreatine can be produced by creatine kinase (CK) after ATP synthesis in mitochondria, while reversal of this reaction by cytoplasmic CK regenerates ATP at sites that do not have mitochondria but still have high energy demands [6]. The neural retina had previously been thought to use glucose exclusively as an energy substrate, but now lipid β-oxidation has also been shown to be an energy substrate for photoreceptors [7]. More mysteries about retinal metabolism are being solved with the development of techniques such as stable isotope-based metabolic analysis with mass spectrometry [8].

### 1.2. Glucose Metabolism

Glucose metabolism in the retina remains incompletely understood. Despite the reliance on oxidative phosphorylation (OXPHOS), the human neural retina (especially the outer retina) metabolizes most of the glucose it consumes through anaerobic glycolysis, preferentially producing lactate instead of generating ATP through oxidative phosphorylation, even in the presence of oxygen [9,10]. This phenomenon, which is called the Warburg effect, is widely observed in cancer cells, which are highly metabolic and proliferative [11,12]. The Warburg effect is thought to promote biosynthesis, provide rapid ATP synthesis, and participate in the generation of reactive oxygen species (ROS) and other pathways in cancers [13]. A high Warburg effect activity in the retina may provide the rapid ATP supply that is required by the synaptic activity required for phototransduction. The increased glucose flux associated with the Warburg effect provides increased carbon units for biosynthesis. For example, 3-phosphoglycerate in the glycolytic pathway provides serine, which actively participates in multiple retinal functions, including RPE phagocytosis, neuronal crosstalk, and reductive currency generation [14]. Increased flux into the pentose phosphate pathway generates increased NADPH, which can be used to maintain redox balance and synthesize lipids in the retina [15]. The retina also possesses a unique shuttle system whereby the retinal pigment epithelium (RPE) transports glucose to the neural retina for aerobic glycolysis while taking in lactate released by the neural retina [16,17].

### 1.3. Lipid Metabolism

Lipids are an important retinal energy substrate. Mitochondria in RPE cells utilize fatty acids (FAs) from phagocytosed lipid-rich photoreceptor outer segments [18]. Glucose had previously been believed to be the only source of energy in the neural retina, with only immunohistochemical evidence of the presence of fatty acid β-oxidation [19]. Fatty acid β-oxidation has been found more recently to contribute to the energy supply of the neural retina [7]. Meanwhile, glucose uptake of the retina may be curbed by the lipid sensor Ffar1, indicating an interaction between glucose and lipid metabolism in the retina [7]. The neural retina can also use β-hydroxybutyrate, a kind of ketone body produced by RPE cells from FAs, as a metabolic substrate [20]. Müller cell-derived lipoproteins are a potential source of lipids for photoreceptors for synapse formation and energy production [21]. 

Lipids are also a major structural component of cell membranes, with the phospholipid bilayer being the fundamental structure of biological membranes. Those phospholipids are primarily glycerophospholipids and phosphosphingolipids. The roles of the sphingolipids will be explained later in the review. The major structural glycerophospholipids that form all eukaryotic biological membranes, including cells within the retina, are divided by their polar heads: phosphatidylcholine (PC), phosphatidylethanolamine (PE), phosphatidylinositol (PI), and phosphatidylserine (PS) [22]. The FA groups vary among different phospholipids, while palmitic acid, stearic acid, oleic acid, and arachidonic acid are present at high levels in the retina [23]. Total PC lipids have been reported to represent about 490 µg per mg of total phospholipids in the retina [23]. PI contains mainly arachidonic acid, while docosahexaenoic acid (DHA) has long been known as the major FA of retinal PS and PE [24]. The biosynthesis of lipids has been intensely studied since their discovery in the retina, describing cholesterol synthesized by the retina as key components of photoreceptor outer segment membranes [25]. The functional significance of very-long-chain fatty acid (VLC-FA) families has recently begun to be understood [26]. Members of this family have been identified in the human retina, predominately in photoreceptor outer segments [27]. 

Lipids also act as second messengers for signal transduction in rod outer segments [28]. Signalling pathways that involve lipid mediators, including phosphoinositide 3-kinase (PI3K), are also affected by retinal lipid metabolism [29]. In the retina, the PI3K/AKT/mTOR pathway participates in cell survival regulation via discrete signalling pathways [30,31,32] and proliferation [33]. 

### 1.4. Amino Acid Metabolism

Amino acids play critical roles in neurotransmission and metabolic homeostasis. Glutamate, an essential excitatory neurotransmitter, can be neurotoxic if dysregulated [34]. Glutamate is synthesized from the tricarboxylic acid (TCA) cycle and can be taken up by Müller cells in the retina, where it enters the glutamate/glutamine neurotransmitter cycling, or is metabolized to be glutathione, which is crucial for retinal redox homeostasis [35]. Aspartate, another excitatory neurotransmitter, can be metabolized from glutamate via transamination and converted to asparagine in the presence of glutamine [36]. γ-aminobutyric acid (GABA) is the main inhibitory neurotransmitter in the retina, participating in the inhibitory feedback of the light response signal [37]. GABA is also a product of glutamate metabolism, synthesized from glial glutamine and capable of contributing to total glutamate/glutamine neurotransmitter cycling [38]. Glycine is another major inhibitory neurotransmitter that is also a substrate for glutathione synthesis [39]. Serine is the precursor of glycine. De novo serine biosynthesis diverges from the glycolytic intermediate 3 phosphate glycerate. This pathway is primarily active in Müller cells and may also influence glutathione synthesis [40]. Proline metabolism in the RPE has recently been reviewed, highlighting its importance in transport and utilization in maintaining retinal metabolism and homeostasis [41]. Other reviews have discussed related receptors and mediators [42,43].

### 1.5. Purine Metabolism

Purines are mainly synthesized by the complementary salvage pathway (recycling hypoxanthine, inosine, and adenine as substrates to generate purine nucleotides), while fewer purines are produced from the de novo biosynthetic pathway, which consumes ATP, glutamine, folate, glycine, aspartate, and carbon dioxide [44]. In the retina, inosine monophosphate dehydrogenase (IMPDH), an enzyme that catalyzes the rate-limiting step in de novo GTP synthesis, has been reported to contribute to the production of the majority of guanine [45]. Inosine and hypoxanthine can be further oxidized into xanthine and uric acid through the purine degradation pathway [44]. Purine metabolism is required to generate DNA and RNA molecules, adenosine triphosphate (ATP), nicotinamide adenine dinucleotide (NAD), and coenzyme A. The products cyclic adenosine monophosphate (cAMP) and guanosine triphosphate (GTP) participate in signalling transduction [46]. ATP functions as a receptor-mediated neuromodulator in the retina. The P1 (adenosine) receptors are G protein-coupled receptors that control multiple retinal functions, including cell proliferation, migration and death of retinal cells, and the modulation of inflammation [47,48]. ATP can also bind to P2 (nucleotide) receptors, including P2X (ligand-gated ion channels) and P2Y (G protein-coupled receptors) receptors [49]. Among the P2 receptors, the P2X7 is the most studied receptor involved in the induction of neuronal and microvascular cell death under pathogenic conditions, including ischemia–hypoxia, elevated intraocular pressure, and diabetes [50]. The cyclic guanosine monophosphate (cGMP) can participate in multiple signalling transductions as a second messenger [51]. In photoreceptors, the synthesis of cGMP is catalyzed by membrane guanylyl cyclase’s (GCs) and regulated by Ca^2+^-binding, and GC activating proteins (GCAPs). Dysregulation of cGMP contributes to photoreceptor death [52].

### 1.6. Other Metabolic Pathways in the Retina

Additional molecules that are actively metabolized include pigments, such as lutein and zeaxanthin, and retinoids. The transport of retinoids, mechanisms by which this chromophore is supplied to both rods and cones, and the metabolism of retinoids within the posterior segment of the eye, are reviewed in detail elsewhere [53]. The knockout of genes involved in retinoid metabolism, such as *Rho* and *Irbp*, in animals resulted in dysregulation of retinal metabolism and photoreceptor degeneration [54]. Unsurprisingly, retinal metabolism is also severely impaired when the blood supply of the choroid and retina is impaired [55]. Such diseases include, but are not limited to, glaucoma, retinal artery or vein occlusion, retinopathy of prematurity, and retinal detachment, reviewed in detail elsewhere [56,57,58,59].

## 2. Method of Literature Research

PubMed was used for the literature search. The keywords used were: “retina, metabolism, lipid, amino acid, glucose, purine, diabetic retinopathy, AMD, IRD, MacTel, gene therapy, oral drug administration, novel therapies”. All relevant articles published between 1 January 2011 to 14 December 2021 were included, with further hand-searching of references from published articles. Only articles written in English were included. ClinicalTrials.gov was used to search for relevant clinical trials, utilizing the keywords: “retinal diseases, gene therapy”.

## 3. Age-Related Macular Degeneration (AMD)

### 3.1. Metabolic Features of AMD

AMD is a leading cause of visual impairment and irreversible vision loss in the elderly population globally [60]. AMD manifests clinically as early AMD (characterized by drusen and retinal pigment epithelial disturbance) and late AMD, which is further differentiated into geographic atrophy (GA) (late “dry” AMD) and exudative AMD (“wet” AMD) [61]. Early AMD is characterized by the formation of deposits containing lipids (drusen, which are used to assess the severity of AMD [62]) beneath the RPE or between the RPE and Bruch’s membrane [63]. Late AMD patients suffer from progressive lipid accumulation in the RPE, with dysfunction of the RPE-Bruch’s membrane–choroidal capillary complex [63,64]. The dysregulation of lipid metabolism, including cholesterol metabolism in RPE, may contribute to the pathogenesis of AMD [65,66]. Oxidative stress in AMD contributes to lipid oxidation and promotes the accumulation of inflammatory molecules generated from microglia and macrophages during drusenogenesis [67]. Unlike other cells in the retina, RPE can also metabolize FAs to produce β-hydroxybutyrate as an alternative retinal energy source. Although there is a global bioenergetic crisis in the RPE that can eventually starve both central and peripheral photoreceptors, the disproportionately high loss of macular photoreceptors may be attributable to the higher density of cones in this region [68]. 

### 3.2. Lipids and Lipoproteins in Peripheral Circulation and Their Relationship with AMD

Associations have been reported between AMD and lipoproteins, including total cholesterol (TC), triglycerides (TG), low-density lipoprotein cholesterol (LDL-C), high-density lipoprotein cholesterol (HDL-C), and HDL, but with conflicting conclusions [69]. A meta-analysis of 19 studies reported that a high HDL-C level was associated with an increased AMD risk, whereas participants with a high TC, LDL-C, and TG concentrations may show a decreased risk for this disease [70]. Table 1 concluded some of the main metabolic pathways and their correlations with AMD.

The Helsinki Businessmen Study (*n* = 209) examined whether serum cholesterol in early middle age is associated with the development of AMD later in life. Serum levels of TC at baseline (1964–1973) were significantly higher in subjects who progressed to intermediate or late AMD, or with drusen ≥125 µm, compared with the rest of the study population. Serum levels of TC, LDL, and TG in the follow-up in 2011 were lower in subjects with AMD than those without, whereas HDL levels showed no significant difference [71]. This study found that a higher TC at baseline predicted the progression of AMD, but that patients with AMD had a lower TC, LDL, and TG during follow up compared with the baseline. Thus, the association between the level of circulating lipids and lipoproteins and the risk of AMD may vary between different stages of the disease [71]. The defects of genes encoding enzymes involved in metabolic pathways of different classes of lipid (ABCA1 and GPX4) and lipoprotein (APOE) have been associated with AMD in a genome-wide association study [72]. A study based on plasma samples from 100 patients with wet AMD and 192 controls (with early AMD) found that multiple long-chain acylcarnitines, which are part of the carnitine shuttle pathway, were significantly increased in wet AMD patients, suggesting that fatty acid metabolism may be involved in the pathophysiology of wet AMD [73].

### 3.3. Lipids and Lipoproteins in Diet and Their Relationship with AMD

Dietary FAs are related to the development of AMD. Systemic biomarkers of lipid intake have been found to be good predictors of retinal lipid content. Eyes from donors at different stages of AMD had significantly decreased serum levels of very long chain-polyunsaturated fatty acids (VLC-PUFAs) and low ω-3/ω-6 ratios [74]. A dose-response meta-analysis reported that increasing the dietary intake of ω-3 PUFAs, specifically DHA and eicosapentaenoic acid (EPA), was associated with a reduced risk of early AMD, while other FAs had no significant association [75]. A prospective cohort study found that higher intakes of EPA and DHA may prevent or delay the occurrence of visually significant “intermediate” AMD [76]. A low dietary intake of ω-3 long-chain (LC)-PUFAs has been reported to be a risk factor for AMD [77]. Unfortunately, AREDS2, a multicentre randomized controlled trial (RCT), reported that supplementation of ω-3 FAs had no significant overall effect on AMD incidence or progression [78]. Another RCT, Vitamin D, and Omega-3 Trial (VITAL), also failed to demonstrate that supplementation of vitamin D3 or marine ω-3 FAs had any significant overall effect on AMD incidence or progression [79]. 

### 3.4. Dietary Glycaemic Index (GI) and Development of AMD

The GI indicates how fast blood glucose levels rise after consuming carbohydrates [80]. GI was reported to be a possible independent risk factor for early age-related macular degeneration [81]. A prospective study from AREDS also reported that a high GI diet was associated with a high risk of progression for AMD patients, especially those at high risk of advanced AMD [82]. The Blue Mountains Eye Study found evidence that supported a high GI diet as a risk factor for developing early AMD [83]. Multiple mechanisms were hypothesized to contribute to the high GI-induced progression of AMD, including increased advanced glycation and lipoxidation end products (AGEs and ALEs), the generation of ROS, and the activation of the polyol, PKC, and hexosamine pathways, as well as the induction of inflammation and apoptosis [84]. 

## 4. Diabetic Retinopathy (DR)

The prevalence of diabetes in adults was estimated to be 8.8% in 2015 and is predicted to rise to 10.4% by 2040, affecting 642 million people [85]. Diabetes affects many parts of the eye, but the primary vision-threatening pathology occurs in the retina [86]. DR affects 1 in 3 people with diabetes and remains the leading cause of blindness in working-age adults [87]. Clinically, DR can be non-proliferative (NPDR) or proliferative (PDR). NPDR represents the early stage of DR, with increased vascular permeability and capillary occlusion while PDR, a more advanced stage of DR, is characterized by pre-retinal neovascularization [88]. The Diabetes Control and Complications Trial was the first to link hyperglycaemia with the development and progression of DR unequivocally [89]. Several biochemical mechanisms have been proposed to explain the retinal vasculopathy and neuropathy that hyperglycaemia causes.

### 4.1. The Interaction between Vascular Dysfunction and Metabolic Dysregulation

One of the major characteristics of DR-related vascular disorders is vasoregression. The dysfunction of the vasculature is accompanied by fluid accumulation in the macular region, which is one of the most common causes of loss of vision in people with diabetes [90]. 

The angiopoietin–Tie system was reported to mediate the hyperglycaemia-induced pericyte and endothelial cell loss [91,92]. The activity of the polyol pathway in endothelial cells and pericytes also contributes to hyperglycaemia-induced vascular disorders. The polyol pathway is a two-step metabolic pathway in which glucose is reduced to sorbitol and converted to fructose. This pathway is activated when there is excessive glucose [93]. Table 2 concluded some of the main metabolic pahtways and their correlations with DR.

NADPH (the main antioxidant in the retina) and NAD^+^ (vital for cellular metabolism) would be consumed in the process [94], which might lead to increased oxidative stress levels in the retina. Sorbitol accumulates intracellularly because it cannot easily penetrate the cellular membrane. One part of the sorbitol molecule is catalyzed in the polyol pathway by sorbitol dehydrogenase, leading to the oxidation of sorbitol to make fructose, which is difficult to process further. Subsequently, sorbitol and fructose can accumulate in cells, increasing osmotic pressure and disrupting membrane permeability [95]. 

The hexosamine pathway may participate in the pathogenesis of diabetic vasculopathy. Hexosamine content is increased in the retinal tissues of humans and rats with diabetes [102]. The modification of O-linked β-*N*-acetylglucosamine (*O*-GlcNAc) is elevated through the hexosamine pathway under hyperglycaemic conditions. The increased *O*-GlcNAc modification of p53, an important tumour suppressor that induces apoptosis, was associated with an increase in its protein levels in retinal pericytes. The post-translational modification of *O*-GlcNAc in p53 and its increased levels may contribute to the selective early loss of pericytes during diabetes [96], which contributes to vascular dysfunction and, therefore, metabolic dysregulation. The interaction between the dysregulation of metabolism and the immune system, particularly leukostasis, which can contribute to retinal vascular pathology, is reviewed elsewhere [103].

### 4.2. The Interaction between Neurodegeneration and Metabolism Dysregulation

Another characteristic of DR is neurodegeneration. Hyperglycaemia is thought to induce neuronal degeneration by several mechanisms. One such mechanism is AGEs, which have been extensively investigated in various ocular tissues and are often elevated during aging and in diabetic subjects compared to nondiabetic control subjects [104]. AGEs are considered to be an organic marker of the glycation process and have been linked to neurotoxicity [105]. AGE-induced glycation of Tau protein was believed to participate in the development of Alzheimer’s Disease (AD) [106]. One of the AGEs, MGO, was reported to induce RPE cell death in a caspase-independent manner, relying on ROS formation, mitochondrial membrane potential loss, intracellular calcium elevation, and the ER stress response [99]. Likewise, excessive activation of the polyol pathway leads to ROS accumulation, which induces oxidative stress in cells and induces neuronal death [95]. 

The activation of the hexosamine pathway can also lead to neurodegeneration by promoting ER stress, lipid accumulation, and inflammatory gene expression [97]. Excessive glucose flux through the hexosamine pathway may direct retinal neurons to undergo apoptosis via perturbation of the neuroprotective effect of insulin, mediated by Akt and via induction of apoptosis, possibly by the altered glycosylation of proteins [98]. 

The pentose phosphate pathway (PPP), one branch of glucose metabolism, is the major pathway that provides retinal reducing power [107]. PPP has been found to have a close relationship to obesity-related insulin resistance and insulin secretion [108], which is vital for the regulation of systemic metabolism. Transketolase (TKT), one of the key enzymes in the PPP, was increased after exposure to hyperglycaemia [109], indicating its active role in the hyperglycaemia-induced reactions. The deficiency of thiamine, the coenzyme of TKT, was reported to be related to neurodegeneration via increased oxidative stress and other mechanisms [110]. TKT converts glyceraldehyde-3-phosphate and fructose-6-phosphate into pentose-5-phosphates and other sugars. Benfotiamine, by activating TKT, inhibits the hexosamine and PKC pathways and decreases the formation of AGEs, thus preventing experimental diabetic retinopathy [100]. The deficiency of glucose-6-phosphate dehydrogenase (G6PD), another key enzyme of PPP, was reported to relate to higher PDR prevalence in type 1 diabetes [101]. Metabolomics studies have provided evidence supporting the involvement of PPP in the development of DR [111,112], together with the ascorbic acid (AA) pathway, which also has an antioxidant effect [113]. These findings indicate that increased oxidative stress caused by the malfunction of the PPP and AA pathways may lead to neurodegeneration in DR. 

The dysfunction of amino acid metabolism may also contribute to the development of neurodegeneration in DR. It was demonstrated in 2002 in a rat model that the function of the glutamate transporter in Müller cells was decreased early in the course of DR but could be restored by a disulfide-reducing agent, suggesting the involvement of oxidative stress [114]. It has since been demonstrated that retinal ganglion cell (RGC) death in DR is mainly caused by glutamate-mediated toxicity through the activation of NMDA receptors. At the same time, the inhibition of NMDA by donepezil was retinoprotective [115]. Metabolomic analysis indicated that the arginine to proline pathway was involved in the development of DR, with dysregulation of the nitric oxide synthase (NOS) pathway. Reduced NO availability led to endothelial cell dysfunction and impaired vasodilation, as well as increased the generation of ROS and reactive nitrogen species (RNS), which accelerate neurodegeneration [116].

### 4.3. Other Metabolic Changes in DR

One study found 126 metabolic features (defined by m/z, retention time, and ion intensity) that differed significantly between plasma samples from 83 DR patients and 90 controls with type 2 diabetes and no retinopathy, with nine significantly changed pathways, the most significant of which related to the metabolism of niacin and amino acids [117]. A total of 151 features distinguished 34 PDR patients from 49 NPDR patients and pathway analysis revealed alterations in the β-oxidation of saturated FAs, fatty acid metabolism, and vitamin D3 metabolism [117]. These findings indicate that the complicated dysregulation of multiple metabolites and signalling pathways, in addition to hyperglycaemia, likely contributes to the pathogenesis of DR. 

## 5. Inherited Retinal Degenerations (IRD)

IRDs are a diverse group of progressive, visually debilitating retinal diseases that can lead to vision loss and total blindness. Mutations in genes critical to retinal function cause progressive photoreceptor cell death and associated vision loss [118]. IRDs are genetically heterogeneous, with at least 280 identified gene defects reported in the database RetNet (https://sph.uth.edu/RetNet/, last accessed on 14 December 2021.) [119]. Among the 280 genes, 69 were identified for retinitis pigmentosa. Genes related to metabolism include *HK1* (Hexokinase 1, catalyzing the first step in glycolysis), *HKDC1* (Hexokinase Domain-Containing 1, the 5th hexokinase, catalyzing the first step in glycolysis), *ACOX* (Acyl-CoA oxidase, the first enzyme of the fatty acid beta-oxidation pathway), *IDH3A* and *IDH3B* (subunits alpha and beta of Isocitrate dehydrogenase, a TCA enzyme that oxidatively decarboxylates isocitrate to α-ketoglutarate), *IMPDH1* (Inosine monophosphate dehydrogenase 1, a rate-limiting enzyme in the de novo synthesis of guanine nucleotides), and *PDE6* (Phosphodiesterase 6, reducing cytoplasmic levels of cGMP in rod and cone outer segments in response to light) [120]. 

Some systemic metabolic disorders have ocular manifestations. An example is long-chain L3 hydroxyacyl -CoA dehydrogenase (LCHAD) deficiency, where retinopathy manifests as a complication. LCHAD and trifunctional protein (TFP) deficiency are caused by mutations in the genes *HADHA* and *HADHB* (Hydroxyacyl-CoA Dehydrogenase Trifunctional Multienzyme Complex Subunit Alpha and Beta). Both genes participate in fatty acid oxidation and are part of fatty acid oxidation disorders. The mechanism of how these mutations contribute to the development of retinopathy is still unknown [121]. Mutation of the gene *ELOVL4* (elongation of very long fatty acids-4), which is the elongase necessary for the biosynthesis of VLCFA, has been found to cause Stargardt-like macular dystrophy [122]. *NMNAT1* (nicotinamide nucleotide adenylyltransferase 1) encodes NMNAT1, a rate-limiting enzyme that generates NAD^+^ both in a biosynthetic pathway from nicotinic acid mononucleotide (NaMN) and in a salvage pathway from nicotinamide mononucleotide (NMN) [123]. Mutations in *NMNAT1* were reported to cause Leber congenital amaurosis (LCA), one of the IRDs [124,125,126]. Mutations in other genes such as *VLDLR* can lead to a systemic syndrome that includes retinopathy, have also been reported [127].

## 6. Macular Telangiectasia (MacTel)

MacTel, also known as idiopathic juxtafoveolar telangiectasia, is an uncommon ocular disease that can lead to legal blindness, with two major types of the disease being recognized [128]. Type 1 is unilateral and accompanied by pronounced retinal serous exudation and oedema [129]. Although Type 1 was believed to be congenital and a mild subtype of Coats’ disease [130], a recent study suggested the pathological mechanism differs from Coats’ disease [131]. MacTel Type 2 is bilateral and associated with minimal macular oedema, despite hyperfluorescence on retinal fluorescein angiographies [132]. MacTel Type 2 is a degenerative disease of the central retina with typical vascular alterations, including mildly ectatic capillaries, blunted venules, and neovascular complexes [133], with limited treatment options [134]. Evidence suggests that the region of macular pigment loss, which indicates the diseased zone, correlates well with Müller cell depletion in this condition [135]. 

### 6.1. Omics Studies in Type 2 MacTel

An early proteomic study compared eye tissue from MacTel type 2 and control patients using the quantitative mass spectrometry technique iTRAQ [136]. The most prominent differences found were within the glycolytic pathway, where eight proteins were reduced in the diseased macula compared with the peripheral retina of the same eye, and ten were also reduced compared to the macula of a control eye [136]. Müller cell-associated proteins, including GFAP, VIM, and GLNA, were also reduced in the diseased macula, which suggests that Müller cell dysfunction and glycolysis are implicated in the pathology of the disease [136]. 

A later genome-wide association study (GWAS) with 476 MacTel type 2 cases and 1733 controls, associated three independent chromosome loci with the disease [137]. These included 5q14.3 (associated with capillary malformation and hereditary benign telangiectasia, surrounded by genes *RASA1*, *MEF2C*, *TMEM161B*, *TMEM161B-AS1*, *LINC102546226,* and *LINC00461*), 2q34 (associated with decreased blood glycine and serine levels, the relevant gene *CPS1*, which encodes carbamoyl phosphate synthetase 1), and 1p12 (the relevant genes include *PHGDH*, which encodes phosphoglycerate dehydrogenase and is associated with blood plasma serine level, and *HMGCS2*, which encodes 3-hydroxymethylglutaryl-CoA synthase and regulates ketogenesis). Subsequently, they found significant differences in blood serum levels of glycine and serine between MacTel cases and controls [137]. Another GWAS study on 1067 MacTel Type 2 patients and 3799 controls was performed and reported in 2021, which identified eight novel genome-wide significant loci, and confirmed all three previously reported loci [138]. The study prioritized 48 genes implicated in serine–glycine biosynthesis, metabolite transport, and retinal vasculature and thickness, indicating a likely causative role of serine and glycine depletion as well as alanine abundance in the pathogenesis of MacTel type 2 [138].

### 6.2. Sphingolipids and Serine Metabolism in MacTel Type 2

An exome sequence analysis study of serine and lipid metabolisms in a pedigree with hereditary sensory and autonomic neuropathy type 1 that also had MacTel Type 2, identified a defective variant in *SPTLC1* encoding a subunit of serine palmitoyltransferase (SPT) [139]. Circulating deoxysphingolipid levels were 84% higher among patients with MacTel type 2, who did not have pathogenic variants affecting SPT, than among unaffected controls. Deoxysphingolipid levels were negatively correlated with serine levels, which were 21% lower than the controls. Reducing serine levels in mice led to increased retinal deoxysphingolipids and compromised electroretinographic responses [139]. Elevated levels of deoxysphingolipids caused photoreceptor-cell death in retinal organoids [139]. The functions of serine and sphingolipids in the retina have since attracted increased attention. The integrated genetic markers for MacTel, vascular, and metabolic traits have been compiled and Mendelian randomization applied to analyze conditional and interactive genome-wide associations [140]. Genetic disruption of serine biosynthesis was found to be a key driver of MacTel type 2 development and progression [140]. 

Serine is involved in the sphingolipid–ceramide balance for both the outer retina and RPE, and the redox balance for the RPE via serine biosynthesis. Perhaps the most vital function of serine metabolism is free radical scavenging in the entire retina via serine-derived scavengers, including glycine and GSH. The retina appears to have an extensive dependency on serine homeostasis. Consequently, any dysregulation in serine metabolism can result in a wide spectrum of retinopathies [14]. 

## 7. Metabolism Related Therapies

### 7.1. Systemic Drug Administration

The major concern for the systemic administration of drugs is that the efficacy of the delivery to the posterior segment of the eye is extremely low. Systemic delivery of many drugs, particularly if they are large or hydrophilic, into the retina is restricted by the blood–retinal barrier (BRB). In addition, the combination of the systemic volume of distribution, the fast systemic and choroidal blood flow rates, hepatic clearance mechanisms, and smaller local clearance mechanisms result in only a brief duration of action in the retina of systemically administered drugs. This demands a high concentration of systemic drugs with an increased risk of side effects. The net effect is a low to medium bioavailability to the retina and vitreous, depending on whether the physical and chemical properties of the drug allow access across the BRB [141]. Retinal DHA and NPD1 levels were increased by systemic Arg–Gln treatment in murine pups with oxygen-induced retinopathy, with reduced preretinal neovascularisation and restoration of the vascular density of the retina [142]. This study suggested that systemic administration can still be used to treat retinal diseases, especially with compounds that are relatively safe such as nutrients.

Another concern for the systemic administration of metabolic therapies is that cancer cells and the neural retina share key metabolic features, so antimetabolic treatments for cancer may affect neural retinal function and health. For example, inhibiting nicotinamide phosphoribosyltransferase expression and suppressing NAD^+^ synthesis to reduce tumour growth has been reported to be successful in various tumour types, but this is unfortunately associated with severe retinal toxicity [143]. While metabolic therapies for cancers are being explored [144], it will be necessary to monitor and thoroughly evaluate their effects on the retina before clinical applications can be considered. Metabolic therapies for retinal diseases should also be evaluated for cancer. 

### 7.2. Gene Therapy

Several unique characteristics of the retina make it a suitable target for translational gene therapy. First, the human eye is relatively small and easy to access, which makes the efficiency of gene delivery relatively high and cost-effective. Second, the compartmentalized nature of the eye reduces the risks of systemic dissemination of gene therapies, minimizing the risk of off-target systemic gene editing. Third, regions of the eye are relatively immune-privileged, which makes exposure to viral vectors generally well tolerated. Last, visual function and retinal anatomy can be monitored after treatment using a range of non-invasive methods, including OCT, psychophysics, and electrophysiology. Surgical procedures may be adapted to preferentially transduce a particular ocular cell type, with minimal risks to patients undergoing surgery [145]. 

A Phase 3 clinical trial for voretigene neparvovec (AAV2-hRPE65v2) in 2017 reported that gene replacement improved the functional vision in patients with RPE65-mediated inherited retinal dystrophy that was previously considered medically untreatable [146]. This gene therapy was approved by the Food and Drug Administration (FDA) to treat patients affected by Leber Congenital Amaurosis Type 2 (LCA2). As such, voretigene neparvovec became the first gene therapy approved for an inherited disease. Currently, dozens of clinical trials on gene therapy for retinal diseases are in progress, with different genes (such as *RS1* for X-linked juvenile retinoschisis, MCO-010 for the production of optogenetic molecules) and different vectors (mainly different serotypes of adeno-associated virus (AAV)) under investigation [147]. The clinical trials include studies that target *PDE6A* (NCT04611503) and *PDE6B* (NCT03328130) to regulate cGMP metabolism, for the treatment of retinitis pigmentosa patients; these trials are currently recruiting patients. As discussed, multiple metabolic genes contribute to the development of MacTel, including *PHGDH* and *SPTLC1* (in serine and sphingolipids metabolism), so it may be of considerable therapeutic value to explore metabolic targets as treatments of this disease based on outcomes discussed above for other retinal diseases.

### 7.3. Potential Targets

Metabolic targets are being explored to treat various retinal diseases. Lipid metabolism is a potential target for the treatment of early AMD: a review of multiple therapies targeting lipid metabolism tested on mice and other models included desipramine (prevents ceramide production), DHA, apolipoprotein mimetics, and statins (reduces endogenous cholesterol synthesis) with promising results [148]. 

Some new treatments for DR that manipulate metabolic activities have been reported recently. Alpha-lipoic acid (ALA), a naturally occurring dithiol micronutrient that acts as a cofactor for mitochondrial enzyme activity, is considered a “universal antioxidant” due to its potent antioxidant activity. ALA prevents DR by inhibiting *O*-GlcNAc transferase and NFκ-B activity and alleviating oxidative stress. It can activate NRF2 and AMPK in RGCs. Clinical trials conducted in pre-retinopathic people with diabetes reported that ALA with genistein and vitamins could protect retinal cells and reduce inflammation [149]. The coenzyme benfotiamine, which targets transketolase, was reported to benefit patients with DR via different pathways, as described above [100]. Benfotiamine was found to alleviate oxidative stress and improve endothelial function in people with type 2 diabetes [150], with good safety and tolerability [151]. Further studies of its effects in patients with DR are needed. 

Specific IRDs are caused by diverse genetic mutations that ultimately cause photoreceptor degeneration. Boosting the regrowth of outer segments of “dormant” photoreceptors could be beneficial for a diverse range of IRDs. Rod-derived cone viability factor (RdCVF) stimulates the rate of aerobic glycolysis by increasing glucose entry into cones [152]. It has been reported that the loss of hexokinase-2 (HK2), a key enzyme in aerobic glycolysis, does not affect photoreceptor survival or structure, but is required for normal rod function [153]. Another study reported that the selective knockdown of HK2 in rods leads to age-related photoreceptor degeneration and retinal metabolic remodelling [154], rendering HK2 a potential target for the treatment of retinal degeneration. Further studies are required to confirm these observations.

## 8. Conclusions

The retina has exceptionally high energy demands to maintain normal function. The dysregulation of retinal metabolism plays a critical role in several retinal diseases, notably AMD, DR, and MacTel type 2. By exploring the relationship between the dysregulation of lipid metabolism and AMD, the dysregulation of glucose metabolism and DR, and the dysregulation of serine and sphingolipid metabolism and MacTel type 2, promising new therapeutic targets may be developed. Although specific gene mutations can cause IRDs, targeting the metabolic system that supports photoreceptors may represent a universal and clinically practical treatment approach.

## Figures and Tables

**Table 1:** Main metabolic pathways and their correlations with AMD.

Metabolic Pathways	Correlations with AMD	References
Total cholesterol (TC) metabolism	High TC level indicates decreased risk of AMD	[70]
	High TC level at baseline predicts risk of progression, but TC level is low in progressed patients	[71]
High-density lipoprotein cholesterol (HDL-C) metabolism	ABCA1: loci for advanced AMD with genome-wide significance	[72]
Lipid hydroperoxides metabolism	GPX4: loci for advanced AMD with genome-wide significance	[72]
The carnitine shuttle pathway	Carnitine shuttle pathway are significantly increased in patients with wet AMD	[73]
Polyunsaturated fatty acids (PUFAs) metabolism	eyes with AMD show decreased levels of very long chain-PUFAs (VLC-PUFAs) and low ω-3/ω-6 ratios	[74]
	high intake of ω-3 PUFAs is associated with a reduced risk of early AMD	[75,76,77]
	ω-3 fatty acids have no effect on AMD incidence or progression	[78,79]

**Table 2:** Main metabolic pathways and their correlations with DR.

Metabolic Pathways	Correlations with Dr	References
Polyol pathway	Contributes to hyperglycaemia-induced dysfunction of endothelial cells and pericytes	[93]
	Consumes NADPH and NAD+ and causes increased oxidative stress	[94]
	Increases osmotic pressure and disrupts membrane permeability	[95]
Hexosamine pathway	Increases O-linked β-*N*-acetylglucosamine modification of p53 and causes loss of pericytes	[96]
	Promotes ER stress, lipid accumulation and inflammatory gene expression and lead to neurodegeneration	[97]
	Perturbs the neuroprotective effect of insulin, mediated by Akt and via induction of apoptosis	[98]
AGEs accumulation	Induces ROS formation, mitochondrial membrane potential loss, intracellular calcium elevation and ER stress response and causes cell death	[95,99]
Pentose phosphate pathway	Inhibits the hexosamine and PKC pathways and decreases the formation of AGEs by boosting transketolase	[100]
	The deficiency of glucose-6-phosphate dehydrogenase is related to higher PDR prevalence in type 1 diabetes	[101]
